# Stroke in a Patient with Tuberculous Meningitis and HIV Infection

**DOI:** 10.4084/MJHID.2013.017

**Published:** 2013-02-18

**Authors:** Maria Bruna Pasticci, Maurizio Paciaroni, Piero Floridi, Enisia Cecchini, Franco Baldelli

**Affiliations:** 1 Infectious Disease Section, Department of Experimental Medicine and Biochemical Sciences, University of Perugia, Perugia, Italy.; 2 Stroke Unit and Division of Cardiovascular Medicine, University of Perugia, Perugia, Italy.; 3 Department of Neuroradiology, Hospital Santa Maria della Misericordia, Perugia, Italy.

## Abstract

Tuberculous meningitis (TBM) is a devastating disease. TBM occurs more commonly in HIV infected patients. The influence of HIV co-infection on clinical manifestations and outcome of TBM is not well defined. Yet, some differences have been observed and stroke has been recorded to occur more frequently.

This study reports on an HIV infected Caucasian female with lung, meningeal tuberculosis and stroke due to a cortical sub-cortical ischemic lesion.

TBM was documented in the absence of neurologic symptoms. At the same time, miliary lung TB caused by multi-susceptible *Mycobacterium tuberculosis* was diagnosed. Anti-TB therapy consisting of a combination of four drugs was administered. The patient improved and was discharged five weeks later.

In conclusion, TBM and multiple underling pathologies including HIV infection, as well as other risk factors can lead to a greater risk of stroke. Moreover, drug interactions and their side effects add levels of complexity. TBM must be included in the differential diagnosis of HIV infected patients with stroke and TBM treatment needs be started as soon as possible before the onset of vasculopathy.

## Introduction

Central Nervous System (CNS) disease due to *Mycobacterium tuberculosis* accounts for 1–10% of all tuberculosis (TB) cases,^1^ mostly affecting children^1^ and HIV infected persons.^1^ Foreign born persons are also more represented.^1^ Meningitis (TBM) is the most frequent presentation of CNS TB, with its most serious consequence being brain infarction.^1^ Most TBM associated brain infarcts are multiple, bilateral, symmetric, located in the basal ganglia, anterior thalamus, anterior limb and the genu of internal capsule.^2^,^3^ Cortical, sub-cortical white matter, brainstem and hindbrain involvement are less common, except in cases prolonged by TB treatment and HIV co-infection.^2^–^6^ Stroke in TBM can be ischemic or hemorrhagic^7^ and secondary to vasculitis or intimal proliferation or both, with or without thrombosis or spasms. Aneurismal dilatation, ruptured mycotic aneurism, granulomatous septic embolism and arteries being strangulated by inflammatory exudates have also been noted.^2^,^3^ Interestingly, ischemic stroke has been reported more frequently in a cohort of patients with a diagnosis of TB not involving CNS over 3 years of follow up, indicating that TB and not only TBM is a potential risk factor for stroke.^8^ Moreover, tuberculous vasculopathy has been described to occur in any organ affected by tuberculosis.^3^

Our case report describes a HIV co-infected Caucasian female who developed stroke due to TBM secondary vasculopathy with brain infarction of the middle cerebral artery.

## Case presentation

A 45 year old Caucasian female with HIV infection, CDC-A3 and HCV, genotype 1b co-infection, Child-Pugh score 5, non-active IV drug habit, on 35 mg day of methadone, smoking habit of 20 cigarettes per day, nutritional disorder that had led recently to a loss of weight >10 kilos and on HIV treatment including tenofovir/emtricitabina and lopinavir/ritonavir was admitted to a local hospital complaining of fever which began a week prior as well as cough over the last 2 days. The patient was taking levofloxacin 500 mg day during the last week. Three months before admission immune-viral assays resulted being: HIV-RNA 71 copies ml, CD4 T 144 mm^3^. HCV-RNA resulted 780.000 copies ml. On admission, the liver was 5 cm below the costal margin, the erythrocyte sedimentation rate (ERS) was 78 mm 1° hour, hemoglobin 10.8 mg dl, white blood cells 4400 mm^3^, neutrophils 79.6%, coagulation, liver and renal functional tests were normal. A chest radiograph depicted micro nodular shadowing consistent with miliary TB. This finding was confirmed with a chest computed tomography (CT). A bronchoscopy did not show lesions. Bronco-alveolar lavage (BAL) fluid was negative for acid fast bacilli (AFB) and *M. tuberculosis* complex strand displacement amplification (SDA) was non-reactive. Culture subsequently identified multi-susceptible *Mycobacterium tuberculosis*. On the same day, in the absence of neurologic symptoms, cerebrospinal fluid (CSF) was examined leading to the diagnosis of a probable TB meningitis:^9^,^10^ glucose CSF 25 mg dl (blood glucose 99 mg dl), white cells 150 mm^3^ and proteins 273 mg dl, Ziehl-Neelsen (ZN), SDA and culture for *M. tuberculosis* resulted negative. CSF cultures for bacteria and fungi were also negative. Latex agglutination test for *Cryptococcus* resulted negative both on CSF and blood. Isoniazid 300 mg day, ethambutol 1200 mg day, pyrazinamide 1500 mg day, rifabutin 150 mg day^11^ and dexamethasone 4 mg^11^,^12^ per day were added to anti-retroviral therapy and levofloxacin. Four days later, the patient developed a right side stroke. A brain CT depicted a left frontal parietal cortical sub-cortical post ischemic high density lesion.

**Figure 1:**
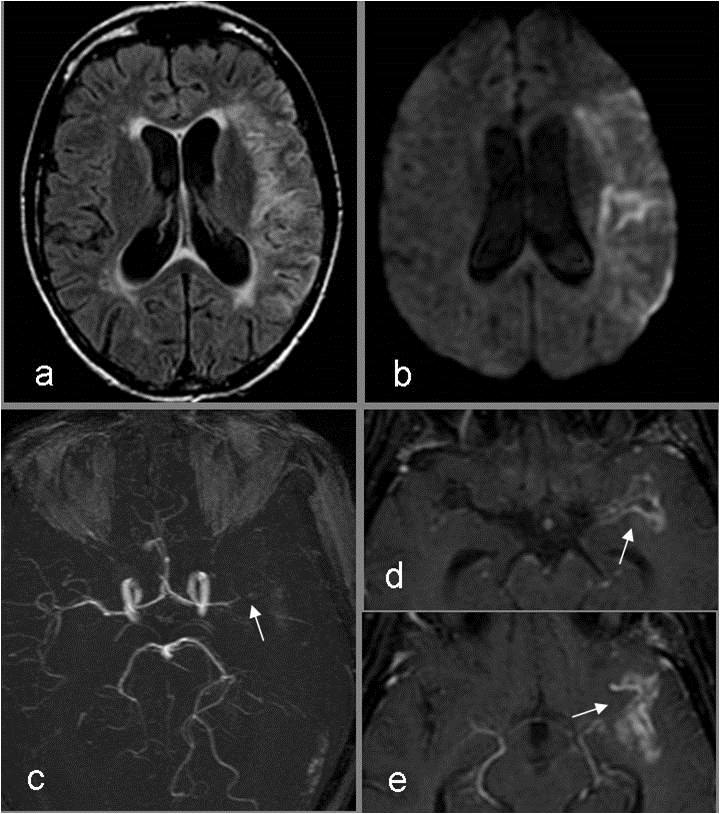
a) MRI fluid attenuated inversion recovery (FLAIR): temporal-parietal left side cortical sub-cortical hyper intense lesion; b) MRI diffusion weighted images (DWI): reduced diffusion consistent with recent ischemia; c) angio MRI: absence of flow through the left middle cerebral artery (arrow), d and e) thickening and contrast enhancement on the left Sylvian fissure surrounding the cerebral artery.

On admission to our Infectious Disease Clinic (IDC), 72 hours later, the patient was aphasic with right hemiplegia (Glasgow coma score non-applicable). Tuberculosis skin (TST) and *γ*-interferon assay (IGRA) tests resulted negative. The repeated CD4 T cells count showed 72 cells mm^3^. The rapid plasma reagin treponemal test for syphilis was negative. Ultrasound of carotid arteries, EKG, trans-thoracic heart ultrasound, antinuclear antibodies, anti neutrophil cytoplasm antibodies, lupus anticoagulant factor and factor V Leiden were negative. Protein S, protein C, anti-thrombin III, homocysteine values were in the normal ranges. A repeated brain CT confirmed the fronto-parietal cortical sub-cortical ischemic lesion with minimal hemorrhagic transformation. Magnetic resonance (MRI) and angio-MRI (Figure 1) also depicted recent ischemic cortical sub-cortical lesion with minimal hemorrhagic transformation, absence of flow through the left middle cerebral artery and thickening as well as contrast enhancement on the left Sylvian fissure surrounding the artery. Thus, the patient was diagnosed with TBM complicated with stroke.^2^–^6^ The patient continued with tenofovir/emtricitabine and lopinavir/ritonavir plus isoniazid 300 mg day, ethambutol 1200 mg day, pyrazinamide 1500 mg day and rifabutin 150 mg day^11^ plus meropenem for the first 7 days, whereas corticosteroids and levofloxacin were suspended. Moreover, due to the presence of multiple risk factors for stroke and the absence of an absolute contraindication, aspirin100 mg day was administered. This was done despite the radiological findings evidenced microbleeds in the ischemic lesion. A percutaneus gastrostomy was performed as a nasal gastric tube was not tolerated. General and neurologic conditions improved while on anti-TB, anti-HIV and aspirin treatments. Two weeks later, CFS findings also improved: glucose 41 mg dl (blood glucose 93 mg dl), white cells 32 mm^3^, all lymphocytes, and proteins 174 mg dl, microscopic and culture for *M. tuberculosis*, bacteria and fungi were negative. Latex agglutination test for *Cryptococcus* test resulted once again negative. Real time polymerase reaction (PCR) for *Toxoplasma gondii*, *Human poliomavirus JC-V*, *Herpes simplex* virus type I and type II, *Herpes* virus type 8, *Varicella Zoster* virus, *Cytomegalovirus* resulted negative on CSF. The test for *Cytomegalovirus* antigen pp65 (Indirect Immunofluorescence, anti-CMV pp-UL83, Argene, Verniolle, France) in blood was also reported being negative.

Five weeks later, the patient was transferred to another hospital where the prescribed treatments and rehabilitation were continued with further improvement.

## Discussion

The patient reported had several risk factors for TB which included HIV infection^1^–^6^ nutritional disorder with a recent weight loss of over 10 kilos, past drug addiction and cigarette habit.^1^ CD4 T lymphocytes <200 mm^3^ were a further risk factor for extra-pulmonary TB.^5^,^6^ Active TB disease can enhance HIV replication and consequently accelerate the destruction of CD4 T lymphocytes and the course of HIV infection.^5^ On admission, only fever, which had been present for 7 days, as well as cough for the past two day were the only clinical manifestations. Chest radiograph suggested miliary TB. This diagnosis was confirmed when multi susceptible *M. tuberculosis* was identified in respiratory secretion. On clinical findings, the patient was immediately administered anti-TB therapy which resulted being effective, based on successive susceptibility test results. The same day, in the absence of neurologic manifestations, CSF was examined evidencing features consistent with probable tuberculous meningitis: pleiocytosis, white cell count 150 cell mm^3^, increased protein level and low glucose concentration plus negative results for bacteria, fungi and virus.^11^

TBM is sub-acute disease and its symptoms are usually present for several days prior to diagnosis. After a period of non-specific signs, such as low grade fever, malaise, headache, dizziness, vomiting or personality changes which can last several weeks, patients can develop more severe headache, altered mental status, stroke, hydrocephalus and cranial nerve deficit.^10^ It has been reported that the risk of TBM is greater in HIV infected patients with TB.^5^,^6^,^9^,^10^,^13^ Fever, severe impaired cognition, focal signs, seizures, lympho-adenopathy and other concomitant TB localizations are more often observed among HIV positive patients with TBM.^3^,^5^,^10^,^13^,^14^ Patients with HIV co-infection and with lower CD4 T cell counts more likely atypically present with more subtle and less specific neurologic manifestations.^5^ The level of immune compromise can also cause differences in neurological imaging and pathology findings with lower levels of inflammatory reaction, reduced exudates and basal meningeal enhancement, less frequent hydrocephalus and higher bacterial counts.^5^ Brain infarction has been reported more commonly in HIV co-infected patients with TBM.^15^ These lesions of vascular origin, which more often occur in HIV infected patients, tend to be localized in the cortical, sub-cortical white matter, brainstem and hindbrain^2^–^7^,^15^ but do not seem to correlate with the severity of inflammation.^3^ It has been hypothesized that *M. tuberculosis* itself can be vasculotoxic or that HIV rather than TB mediated vascular damage can also be the cause.^3^ Several studies have reported on an increased risk of stroke in HIV infected patients. 16–18 Moreover, in our patient, other factors for stroke were present: cigarette smoking, past drug addiction^19^ and compensated chronic HCV infection.^20^ These all may have contributed to the development of cerebral vascular disease, nevertheless, the more likely diagnosis was TB secondary vasculopathy given that the patient was HIV co-infected with a low CD4+T lymphocyte count, miliary TB, CSF abnormalities with negative results for other microorganisms and brain radiologic findings congruent with those of tuberculous meningitis.^1^–^9^,^21^–^24^ Both CT and angio-MRI detected the ischemic lesion,^1^,^3^ and diffusion weighted images (DWI) allowed to define the timing of the lesion.^23^ Hemorrhagic transformation has also reported in patients with TBM.^3^,^7^,^23^ In our case it was not a contraindication for the administration of aspirin. MRI showed gadolinium enhancement of the left Sylvian fissure surrounding the middle cerebral artery.^1^,^3^ Cerebrospinal fluid examination is the cornerstone of TBM diagnosis and the gold standard is the identification of *M. tuberculosis* in the CSF.^1^,^5^,^9^–^11^ The latter of these two requires considerable amount of time but anti-TB treatment needs to be started as soon as possible. Being so, most cases are treated before and or without microbiological diagnosis.^1^–^3^,^5^–^6^,^9^–^11^

The value of anti-inflammatory treatment with corticosteroids^9^–^11^ in preventing infarction in TBM is controversial and the role on anti-TB therapy on the development of stroke is also not well defined.^3^,^20^ Misra et al. in a prospective, randomized study, in 2010, reported a reduction rate of stroke and mortality in a subgroup of patients with TBM randomized to receive 150 mg of aspirin daily.^24^ Our patient was admitted in 2009, before the study by Misra. Aspirin was prescribed because the patient had several other risk factors for stroke and the microbleeding was not a contraindication to the administration of aspirin.

In conclusion, TBM and multiple underling pathologies including HIV infection, as well as other risk factors can lead to a greater risk of stroke. Moreover, drug interactions and their side effects add levels of complexity. TBM must be included in the differential diagnosis of HIV infected patients with stroke and TBM treatment needs be started as soon as possible before the onset of vasculopathy.
